# Evolving landscape of PD-L2: bring new light to checkpoint immunotherapy

**DOI:** 10.1038/s41416-022-02084-y

**Published:** 2022-12-15

**Authors:** Yuqing Wang, Jiang Du, Zhenyue Gao, Haoyang Sun, Mei Mei, Yu Wang, Yu Ren, Xuan Zhou

**Affiliations:** 1 Department of Maxillofacial and Otorhinolaryngological Oncology, Tianjin Medical University Cancer Institute and Hospital; Key Laboratory of Cancer Prevention and Therapy, Tianjin Cancer Institute; National Clinical Research Center of Cancer, 300060 Tianjin, China; 2 Department of Cell Biology, School of Basic Medical Sciences, Tianjin Medical University, 300070 Tianjin, China; 3 Department of Genetics, School of Basic Medical Sciences, Tianjin Medical University, 300070 Tianjin, China

**Keywords:** Tumour immunology, Head and neck cancer, Tumour immunology

## Abstract

Immune checkpoint blockade therapy targeting programmed cell death protein 1 (PD-1) has revolutionized the landscape of multiple human cancer types, including head and neck squamous carcinoma (HNSCC). Programmed death ligand-2 (PD-L2), a PD-1 ligand, mediates cancer cell immune escape (or tolerance independent of PD-L1) and predicts poor prognosis of patients with HNSCC. Therefore, an in-depth understanding of the regulatory process of PD-L2 expression may stratify patients with HNSCC to benefit from anti-PD-1 immunotherapy. In this review, we summarised the PD-L2 expression and its immune-dependent and independent functions in HNSCC and other solid tumours. We focused on recent findings on the mechanisms that regulate PD-L2 at the genomic, transcriptional, post-transcriptional, translational, and post-translational levels, also in intercellular communication of tumour microenvironment (TME). We also discussed the prospects of using small molecular agents indirectly targeting PD-L2 in cancer therapy. These findings may provide a notable avenue in developing novel and effective PD-L2-targeted therapeutic strategies for immune combination therapy and uncovering biomarkers that improve the clinical efficacy of anti-PD-1 therapies.

## Introduction

Immune checkpoint blockade therapies targeting programmed cell death protein 1 (PD-1) achieve marked clinical benefits in cancer patients harboring programmed death ligand-1 (PD-L1) expression tumours [1–6]. As an indication of KEYNOTE-048, the recurrent/metastatic head and neck squamous carcinoma (R/M HNSCC) patients with a higher combined positive score (CPS > 20) showed a favourable response to anti-PD-1 therapy [3]. It is interesting that anti-PD-1 therapy was also observed in patients with PD-L1 negative expression tumours, including lung squamous cell carcinoma (LUSC) and renal cell carcinoma (RCC) [7–10]. Therefore, effective predictive biomarkers for PD-1 blockade are urgently needed.

Programmed death ligand-2 (PD-L2), another ligand of PD-1, was initially discovered to be expressed in macrophages and dendritic cells (DCs). Recent studies demonstrated that PD-L2 is highly expressed in human cancers, including HNSCC [11], LUSC [12], RCC [13], pancreatic ductal adenocarcinoma (PDAC) [14], and cervical cancer [15]. PD-L2 expression is an independent predictor for progression-free survival (PFS) and the clinical response to pembrolizumab in HNSCC [11]. Together with PD-L1, PD-L2 status should be considered to predict the efficiency of anti-PD-1 immunotherapy.

In this review, we summarised the PD-L2 expression and its immune-dependent and independent function in HNSCC and other solid tumours. We focused on recent findings on the mechanisms that regulate PD-L2 expression and their potential prospective in cancer therapeutics.

## PD-L2 expression pattern in human cancer

**Fig. 1:**
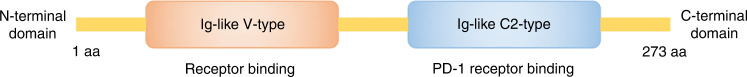
Linear diagrammatic views of functional motifs of PD-L2. The functional motifs of programmed death ligand-2 (PD-L2) contain an Ig-like V-type for receptor binding and an Ig-like C2-type that interacts with programmed death ligand-1 (PD-1). The total amino acids are indicated.

PD-L2 (B7-DC, CD273), a member of the B7 family, is a type I transmembrane protein that contains an immunoglobulin (Ig)-like V-type domain and an Ig-like C2-type domain in its extracellular region (Fig. 1). PD-L2 was expressed not only in tumour cells but also in immune cells, and its high expression has been proven to play an important role in tumorigenesis and immune escape. PD-L2 overexpression indicated a poor prognosis in HNSCC, adenoid cystic carcinoma (ACC) [16], and oesophageal cancer [17]. Specifically, among HNSCC patients, an increased expression of PD-L2 was found positively related to poor relapse-free survival (RFS), progression-free survival (PFS), and OS [11, 18]. Moreover, there is a study on colon cancer showed that PD-L2 expression was positively associated with neuroinvasion and negatively related with CD8 TIL density [^+^^19^].

### PD-L2 in tumour cells

Aberrantly expressed PD-L2 significantly contributes to tumorigenesis and cancer progression due to its function of avoiding the recognition and subsequent killing by the immune system in tumour cells. According to published data, PD-L2 was confirmed highly expressed in HNSCC, salivary gland cancer (SGC), prostate cancer, gastric cancer, colorectal cancer, oesophageal adenocarcinoma, and bladder cancer. And our study also showed that PD-L2 was detected in 62.7% of HNSCC tumours, more than twice that of PD-L1 [18]. In addition, the higher expression of PD-L2 than PD-L1 has also been found in other tumour types [20]. This result may explain why some PD-L1-negative patients still benefit from anti-PD-1 therapy.

### PD-L2 in immune cells

Apart from cancer cells, PD-L2 has significant characteristics in immune cells, such as T cells and NK cells. And these immune cells do not suppress tumours but help them to survive. The PD-L2 is expressed in tumour-infiltrating immune cells (TIICs), CD4 T cells, CD8 T cells, and NK cells, in adenoid cystic carcinoma (ACC) [^+^^+^^21^]. More importantly, PD-L2 expression is also upregulated on tumour-associated macrophage (TAM), and its immune evasion effects become evident when PD-L1 function is dampened in colon carcinoma [22].

## Functions of PD-L2

Although the structure of PD-L2 is similar to PD-L1, the binding affinity between PD-L2 and PD-1 is two- to sixfold higher than that with PD-L1, suggesting PD-L2 is an important molecule in immune escape as the strong interaction inhibits cytokines secretion and proliferation of T cells. Instead of its immune function, PD-L2 was also validated to have a robust biological role during tumorigenesis by interacting with other proteins despite PD-1, such as promoting invasion and triggering chemoresistance of human cancers. These findings open a new era to understand the function of PD-L2 and provide new insights for improving the efficiency of anti-PD-1 therapy.

### Immunosuppressive function of PD-L2

To be a ligand of PD-1, PD-L2 mediates immunosuppressive function. PD-L2 was overexpressed in advanced CRC and correlated with CD8 T-cell exhaustion, suggesting that PD-L2 dysfunction was responsible for the progression of advanced cancers with high proliferation capacity [^+^^19^]. Our published data showed glycosylated PD-L2 decreased cytotoxic T lymphocyte (CTL) in HNSCC [23], which was also proved in GC [24]. In addition, a potential super-enhancer (PD-L1-L2-SE) promoting PD-L1 and PD-L2 expression was found to promote immune escape in breast cancer [25]. In addition, the expression of PD-L2 might affect the therapeutic effect of anti-PD-1 in clear cell renal cell carcinoma and upper-tract urothelial carcinoma [26, 27].

### Other function of PD-L2

In the process of tumour metastasis, epithelial–mesenchymal transition (EMT) is an essential process of tumour cells for migration and colonization, which is determined by the characteristics and plasticity of tumour cells [28, 29]. Much research demonstrated that EMT is strongly related to the level of PD-L2 in HNSCC and other solid tumours. The research showed that PD-L2 participated in the tumour migration process through transportins regulated the Wnt/β-catenin pathway to activate the EMT process (the specific mechanism on the GOLT1B section) [30]. Consistently, PD-L2 expression induced EMT by inactivating the RhoA-ROCK-LIMK2 axis in osteosarcoma [31].

Apart from PD-L2’s role in the metastasis process, our previous study demonstrated that PD-L2 contributes to drug sensibility during treatments. Specifically, glycosylated PD-L2 combine with epidermal growth factor receptor (EGFR) in the cytomembrane to suspend the effect of its inhibitor, cetuximab, in HNSCC (the specific mechanism on the post-translational regulation section) [23].

## Regulatory mechanism of PD-L2

**Fig. 2:**
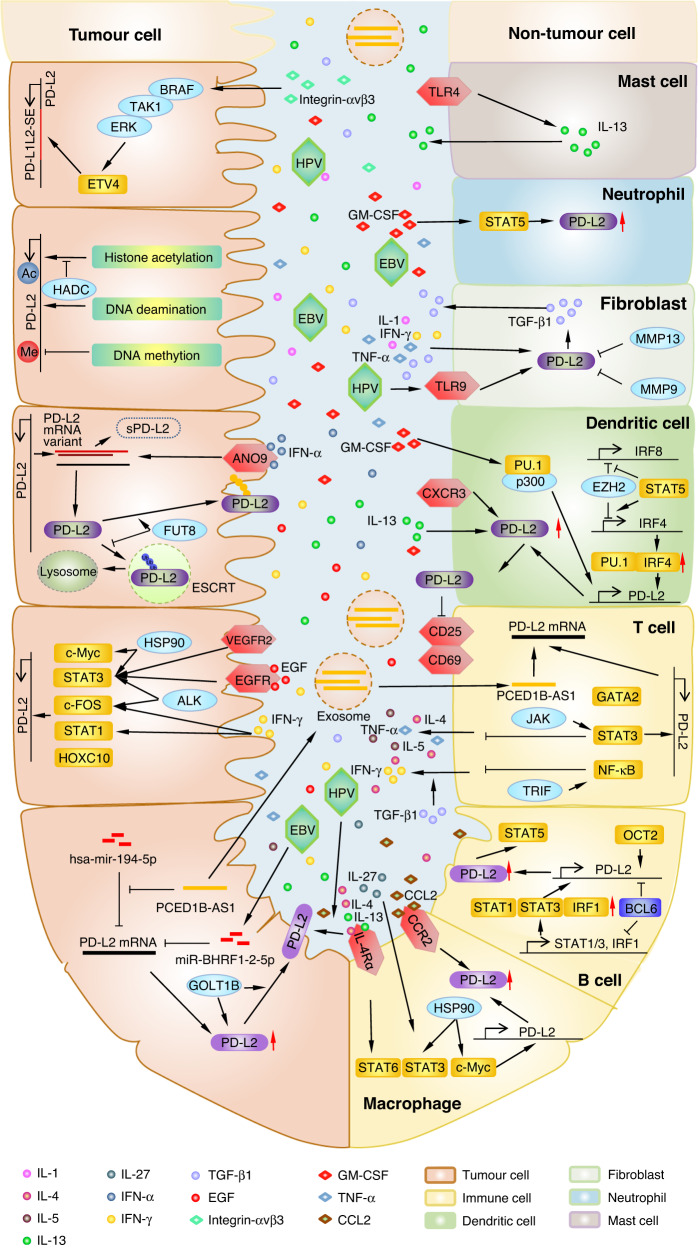
Regulatory crosstalk of PD-L2 in cancer. The role of programmed death ligand-2 (PD-L2) in the maintenance of the tumour microenvironment (TME) and the potential mechanisms of PD-L2 that may contribute to tumour development is shown. PD-L2 is a key factor in tumour progression and immune responses. PD-L2 expression can not only be regulated by the pathway network in a single cell but can also be modulated by cell factors in the TME that form a complex network between different cells.

Based on the crucial roles of PD-L2 in immune activity and tumour progression, a depth understanding of PD-L2 regulation, including genomic alteration, epigenetic modification, transcriptional regulation, post-transcriptional modification, translational regulation, and post-translational modification (Table 1 and Fig. 2), may provide promising combination strategies for predicting and improving PD-1 blockage efficiency.

**Table 1:** PD-L2-regulatory network in tumour cells and immune system.

Regulation type	Effector	PD-L2 expression	Cancer/cell type	Regulation mechanism	Biological behaviour	Refs.
Genomic regulation	JAK2	Upregulation	Non-small cell lung cancer	The amplification of chromosome 9P24.1 increases PD-L2 through JAK2/STAT3 signaling.	Prevented histocompatibility complex class I antigen presentation pathway	[32, 92]
Epigenetic regulation (DNA methylation)	NA	Downregulation	Thyroid carcinoma, gliomas, colorectal cancer, melanoma, gastric adenocarcinomas	PD-L2 DNA promoter methylation decreases PD-L2 mRNA expression in tumour cells.	Promoted Crohn’s-like lymphoid reaction and overall lymphocytic reaction	[36, 37, 93, 94]
Epigenetic regulation (histone acetylation)	p300	Upregulation	Bone marrow dendritic cell	GM-CSF stimulated p300 combines with PU.1 to acetylate PD-L2 histone in DCs.	Suppressed DC-mediated immune response	[40]
	HDAC	Downregulation	Melanoma	HDAC deacetylates PD-L2 histone.	Decreased effectiveness of PD-1 immunotherapy	[41]
Transcriptional regulation	MYC, STAT3	Upregulation	Colon cancer, lung squamous carcinoma, macrophages	HSP90-mediated c-Myc and STAT3 bind with PD-L2 promoter to active transcription.	Stimulated T-cell-mediated tumour clearance	[43]
	BCL6, STAT1, STAT3, IRF1	Downregulation	B cell	BCL6 combines with the PD-L2 promoter to inhibit transcription and binds with STAT1, STAT3, and IRF1 promoters to inhibit transcription and damage their capability of activating PD-L2.	Sustained Tfh and Tfr cell-mediated humoral immunity	[46]
	STAT1, STAT3	Upregulation	Oral squamous cell carcinoma	Cisplatin‐induced STAT1, and STAT3 combine with the PD-L2 promoter.	Promoted proliferation and invasion of cisplatin-resistance	[48]
	STAT1, STAT3, c-FOS	Upregulation	Non-small cell lung cancer	IFN-γ-stimulated STAT1/3, c-FOS or EGFR and EML4-ALK activated STAT3, c-FOS combine with PD-L2 promoter.	Suppressed immune response	[47]
	STAT3	Upregulation	Macrophage	IL-27-stimulated STAT3 increases PD-L2 expression.	Increased tumour-associated macrophages mediated immune suppression microenvironment	[50]
	STAT3, NF-κB	Upregulation	γδT cell	JAK/STAT3 and TRIF/NF-κB axis promote PD-L2 expression and decrease IFN-γ, and TNF-α secretion.	Facilitated intrahepatic recurrence, dissemination and lung metastasis	[49]
	STAT3	Upregulation	Oral squamous cell carcinoma	VEGFR2 mediated STAT3 increasing PD-L2 expression.	Increased tumour migration and invasion	[51]
	STAT5, IRF4	Upregulation	Dendritic cell	STAT5b recruits EZH2 from the IRF4 promoter to the IRF8 promoter and increased IRF4 promotes PD-L2 expression.	Promoted DC-mediated immune response and limited autoimmune development	[40, 52]
	STAT5	Upregulation	Neutrophil	GM-CSF-stimulated STAT5 phosphorylation promotes PD-L2 expression in neutrophils to suppress T-cell proliferation.	Suppressed neutrophil-mediated immune response	[54]
	STAT5	Upregulation	B-1a cell	PD-L2 mediates STAT5 expression to decrease B-1a cell differentiation to ASC and IL-5 secretion in T cells.	Sustained humoral immunity	[53]
	STAT6	Upregulation	Macrophage	IL-4-activated IL-4Rα promote STAT6 that increase PD-L2 expression.	Suppressed macrophage-mediated immune response	[55]
	STAT6	Upregulation	Macrophage	IL-4/IL-13 activated STAT6 promotes PD-L2 expression to convert macrophage to AAM.	Suppressed macrophage-mediated immune response	[56]
	IRF4	Upregulation	Bone marrow dendritic cell	IRF4 combine with PD-L2 promoter with PU.1.	Suppressed DC-mediated immune response	[40]
	GATA2	Upregulation	Glioma	GATA2 combines with PD-L2 promoter region in neoantigen-specific T cells.	Suppressed T-cell-mediated immune response	[62]
	HOXC10	Upregulation	Glioma	HOXC10 combine with PD-L2 promoter.	Promoted proliferation, invasion, and immunosuppression of Glioma	[64]
	OCT2	Upregulation	B cell	OCT2 combine with PD-L2 promoter in B cells.	Suppressed T-cell-mediated immune response	[68]
	ETV4	Upregulation	Breast cancer	Integrin αvβ3 stimulated BRAF/TAK1/ERK axis active ETV4 to combine with PD-L1–L2-SE in breast cancer cells.	Suppressed T-cell-mediated immune response	[23]
Post-transcriptional regulation	PCED1B-AS1	Upregulation	Hepatocellular carcinoma	PCED1B-AS1 sponge hsa-mir-194-5p from PD-L2 mRNA to increase early apoptotic and decrease IL-2 secretion of T cells.	Suppressed immune response	[69]
	miR-BHRF1-2-5p	Downregulation	Diffuse large B-cell lymphoma	MiR-BHRF1-2-5p of EBV combines with PD-L2 mRNA 3’UTR to block PD-L2 expression.	EBV driving B-cell differentiation	[84]
Post-translational modification	FUT8	Upregulation	Head and neck squamous cell carcinoma	FUT8 promotes PD-L2 glycosylation to decrease ubiquitination degradation and increasing membrane expression.	Increased resistance of cetux monotherapy	[21]
Cell communication	LPS/CXCR3	Upregulation	Bone marrow dendritic cell	CXCR3 increase PD-L2 expression to activate DCs development and suppress antigen-specific T cell activation.	Suppressed DC-mediated immune response	[72]
	FCP/TLR4	Upregulation	Bone marrow dendritic cell	TLR4 in mast cells induced IL-13 promote PD-L2 expression in DCs to promote Th2 cell reactivity.	Sustained Th2 cell-mediated humoral immunity	[75]
	CCL2/CCR2	Upregulation	Oesophageal squamous cell carcinoma	The CCL2-CCR2 axis promotes PD-L2 expression in TAMs to increase TAM accumulation, and infiltration and deplete the antitumor effector of T cells.	Promoted immune infiltration and tumorigenesis	[73]
	IFN-α/ANO9	Upregulation	Gastric cancer	IFN-α induced ANO9 increase PD-L2 expression.	Promoted proliferation, migration, invasion, and mediating apoptosis	[74]
Others	GOLT1B	Upregulation	Colorectal cancer	GOLT1B combines with PD-L2 to increase membrane PD-L2 expression to increase apoptosis of T lymphocytes.	Promoted migration and immune escape	[28]
	MMP9, MMP13	Downregulation	Foreskin fibroblast	MMP9/13 combine with PD-L2 and hydrolyse PD-L2 to increase apoptosis of T cells.	Promoted T-cell-mediated immune response	[76]
	TLR9	Upregulation	Head and neck squamous cell carcinoma	HPV stimulated TLR9 increasing PD-L2 expression in fibroblasts and macrophages.	Suppressed immune response	[81]

*OCT2* octamer binding protein 2, *PD-L2* programmed death ligand-2, *ETV4* ETS variant 4, *BRAF* v-RAF murine sarcoma viral oncogene homologue B1, *TAK1* TGF-β-activated kinase 1, *ERK* extracellular signal-regulated kinase, *PD-L1 L2-SE* programmed death ligand-1/2 super-enhancer, *APOBEC3* apolipoprotein B editing catalytic subunits protein 3, *IFN-γ* interferon-gamma, *GM-CSF* granulocyte–macrophage colony-stimulating factor, *DC* dendritic cell, *PU.1* purine rich box-1, *HDAC* histone deacetylase, *MYC* MYC proto-oncogene, *STAT3* signal transducer and activator of transcription 3, *HSP90* heat shock protein 90, *BCL6* B-cell lymphoma 6, *STAT1* signal transducer and activator of transcription 1, *IRF1* interferon regulatory factor-1, *c-FOS* c-fos proto-oncogene, *EGFR* epidermal growth factor receptor, *EML4-ALK* echinoderm microtubule-associated protein-like 4 gene-ALK variant, *EGF* epidermal growth factor, *FUT8* fucosyltransferase 8, *IL-27* interleukin 27, *NF-κB* nuclear factor-κB, *JAK* Janus kinase, *TRIF* TIR domain-containing adaptor inducing interferon-β, *TNF-α* tumour necrosis factor alpha, *VEGFR2* vascular endothelial growth factor receptor-2, *STAT5* signal transducer and activator of transcription 5, *IRF4* interferon regulatory factor-4, *STAT5b* signal transducer and activator of transcription 5b, *EZH2* enhancer of zeste homologue 2, *IRF8* interferon regulatory factor-8, *ASC* apoptosis-associated speck-like protein containing CARD, *IL-5* interleukin 5, *STAT6* signal transducer and activator of transcription 6, *IL-4* interleukin-4, *IL-4Rα* interleukin-4 receptor alpha, *IL-13* interleukin 13, *AAM* alternatively activated macrophages, *GATA2* hematopoietic transcription factor, *IL-2* interleukin 2, *HOXC10* homeodomain‑containing gene 10, *PCED1B-AS1 lncRNA* PC-esterase domain-containing 1B antisense RNA 1, *EBV* Epstein–Barr virus, *MMP9* matrix metalloproteinase 9, *MMP13* matrix metalloproteinase 13, *ANO9* anoctamin 9, *IFN-α* interferon-alpha, *GOLT1B* Golgi vesicle transporter 1B, *CXCR3 C-X-C* motif chemokine receptor 3, *CCL2* C-C motif chemokine ligand-2, *CCR2* C-C motif chemokine receptor-2, *TAM* tumour-associated macrophages, *TLR4* Toll-like receptor 4, *IL-13* interleukin 5, *TLR9* Toll-like receptor 9, *HPV* human papillomavirus.

### Genomic modulation

#### Genomic amplification and mutation

Mutations in cell proliferation caused copy number amplification (CNA) of PD-L2, leading to the upregulation of gene expression levels, which occurred in the tumorigenesis of several solid tumours [15]. Barrett et al. revealed that a high amplification of 9p24.1 (where PDCD1LG2 residues) was detected in triple-negative breast cancer (12/41), colon carcinomas (2/68), and glioblastomas (2/44). PD-L1–L2-SE, which is noted as a super-enhancer located among CD274 and CD273 genes, significantly activates PD-L2 transcription and immune activity.

The amplification of 9p24.1 can improve the level of PD-L2 chromosome fragment through Janus kinase 2 (JAK2)/signal transducer and activator of transcription 1 (STAT1) signaling pathway [32]. In addition, 9p24.1 copy number amplification is related to the increase of JAK2 and phosphorylated STAT3, which is also observed in tumour specimens from 10 recurrent or refractory Hodgkin’s lymphoma patients [33].

Clusters of enhancers (referred to as super-enhancers) drive high-level transcription of oncogenes in squamous cell carcinoma (SCC) [34]. PD-L1–L2-SE, which is noted as a super-enhancer located among CD274 and CD273 genes significantly activates PD-L1 and PD-L2 transcription. Thus, PD-L1–L2-SE-deficient cancer cells are incapable of immune escape and are sensitive to cytotoxicity caused by T cells [25].

#### Epigenetic regulation

In addition to genomic regulation, PD-L2 can also be regulated by epigenetic modification of chromatin without changing its DNA sequence. Till now, DNA methylation and histone acetylation has been proven to be the main epigenetic regulation to modify PD-L2 expression. Immunorecognition can be restored by targeting epigenetic proteins in combination with immune checkpoint inhibitors, thus improving clinical response rates.

#### DNA methylation/demethylation

Bioinformatics analysis indicates that DNA methylation level was related to the prognosis of HNSCC [35]. Methylation level in the PD-L2 promoter region can eventually weaken the PD-L2 protein level through transcriptional inhibition. PD-L2 mRNA expression has been negatively correlated with promoter methylation in thyroid carcinoma and other cancer types, which partly contributes to promoting overall lymphocytic reaction [36, 37]. The EBV infection raises the methylation level of the PD-L2 promoter, thus, regulating the PD-L2 expression of tumours, which causes CD8 T-cell exhaustion and DC infiltration. Therefore, causing poor prognosis in gastric adenocarcinomas and forecasting melanoma patients’ response to immune checkpoint therapies [^+^^38^].

#### Histone acetylation/deacetylation

Histone acetylation alters contacts between histones and nucleosomal DNA, and therefore, increases transcription. High levels of histone acetylation in gene promoter regions are associated with PD-L2 mRNA expression elevation. Histone acetyltransferases (HATs) and histone deacetylases (HDACs) contribute to the regulation of histone acetylation of PD-L2, which can be potential targets in immunotherapy [39]. HAT p300, which interacts with the transcription factor PU.1, promotes H3K27 acetylation in the promoter region of PD-L2 [40]. Moreover, the application of HDAC inhibitors, such as LBH589 (Panobinostat), MGCD0103 (Mocetinostat), MS275 (Entinostat), and PXD101, leads to a more relaxed chromatin state in the PD-L2 promoter regions, thus upregulating the expression of PD-L2. Combining PD-1 immunotherapy, HDAC inhibitor (LBH589) usage, and PD-1 blockade can globally improve survival (>37 days) in C57BL/6 mice, compared to the use of PD-1 blockade alone (30.5 days) [41].

### Transcriptional regulation

Many transcription factors can bind to enhancer-promoter regions of PD-L2 during transcriptional activation. Since PD-L2’s critical role in the immune escape process, amounts of small molecular drugs targeting transcription factors of PD-L2 serve as beneficial strategies with therapeutic potential and have already been used in the clinical treatment of both HNSCC and other solid tumours.

#### Myc

Myc is a well-studied activator of transcription and can promote transcription to modulate tumorigenesis, cancer proliferation, and metastasis in HNSCC [42]. Myc can bind to PD-L2 promoter regions, which promotes the transcriptional activation of PD-L2 in non-transformed macrophages and colon adenocarcinoma and thus, stimulates T-cell-mediated tumour clearance. And Ganetespib inhibitor heat shock protein 90 (HSP90), upstream of Myc, significantly deduces PD-L2 expression [43].

#### BCL6

The binding between B-cell lymphoma 6 protein (BCL6) and genome can recruit diverse chromatin-modifying co-repressor complexes to inhibit gene expression, in order to promote tumorigenesis and cancer progression, such as DNA damage sensing and proliferation checkpoints [44]. A BCL6 mutation has been detected in HNSCC and has been correlated with HNSCC tumour progression [45]. BCL6 negatively regulates PD-L1 and PD-L2 by transcription suppression in germinal center B cells. Furthermore, the binding of BCL6 with introns can suppress PD-L2 transcription mediated by the BCL6 zinc finger structure in its DNA-binding domain. Additionally, BCL6 indirectly regulates PD-L2 expression of B cells dependent on STAT1/3 or IFN regulatory factor-1 (IRF1) expression by binding to their promoter regions, thus indirectly inhibiting PD-L2 of B cells. Therefore, it can maintain the population of T follicular helper (Tfh) cells and T follicular regulatory (Tfr) cells to mediate humoral immunity [46].

#### STAT family

STAT members as a transcription factor family can bind with the PD-L2 promoter region to activate PD-L2 transcription. In non-small cell lung cancer, STAT1 as a response factor to IFN-γ promotes PD-L2 expression, this process can be inhibited by STAT1 inhibitor- Fludarabine [47, 48].

STAT3, as an oncogenic gene, acts on an extensive range of cell types, including tumour cells and immune cells. On the one hand, STAT3 directly combines with PD-L2 promoter regions to activate its transcription [47] and indirectly promotes PD-L2 protein expression by transcription activating of PD-L2 N-glycosyltransferase fucosyltransferase-FUT8 [23]. On the other hand, STAT3 transcriptional actives PD-L2 in multiple immune cells, including B cells [46], T cells [49], and macrophages [50]. Subsequently, Xu et al. found that in γδT cells, the activated JAK/STAT3 signaling pathway can promote the expression of PD-L2 after exposure to indomethacin, and can decrease IFN-γ expression, which facilitates intrahepatic recurrence, intrahepatic dissemination, and lung metastasis [49]. In infiltrating macrophages, the cytokine IL-27 (heterodimer of IL-27B and IL-27Bp28) can activate STAT3 and induce PD-L2 expression, which increases TAM-mediated immune suppression in the TME [50]. Direct inhibiting STAT3 can block PD-L2 through STAT3 inhibitors- cryptotanshinone [48] and Stattic [23]. Moreover, Apatinib inhibits the upstream of STAT3, vascular endothelial growth factor receptor-2 (VEGFR2), to decrease PD-L2 expression through the RhoA-ROCK-LIMK2 axis, eventually, suppressing tumour migration, invasion, and cytoskeletal rearrangement in osteosarcoma [51]. Other STAT3 upstream inhibitors can regulate PD-L2 expressions, such as Erlotinib, epidermal growth factor receptor (EGFR) TKI, and Alectinib anaplastic lymphoma kinase (ALK) TKI [47].

STAT5 has two different subunits, STAT5a and STAT5b, both can function as transcription factors. STAT5b recruit enhancer zeste 2 polycomb repressive complex 2 (EZH2) to methylate IFN regulatory factor-8 (IRF8) promoter region and silence its expression, which causes IRF4 expression increase by EZH2 and IRF4 dissociating and thus, remove IRF4 silence. IRF4 can upregulate PD-L2 expression in bone marrow-derived DCs to promote DC-mediated immune responses and attenuate autoimmune development [40, 52]. Stimulated by the granulocyte–macrophage colony-stimulating factor (GM-CSF), STAT5 can increase PD-L2 expression in neutrophils. Blocking STAT5 by MDK-6314, an inhibitor of STAT5, can impede the differentiation effectiveness of B-1a cells to antibody-secreting cells (ASC) and decrease IL-5 secretion in T cells, which is induced by PD-L2 [53]. Also, another STAT5 inhibitor, STAT5-IN-1, promoted T-cell proliferation [54].

STAT6 is indispensable for IL-4/IL-13/IL-4Rα regulated PD-L2 expression in inflammatory macrophages [55]. Consequently, macrophages are converted to alternatively activated macrophages during Th2-related immunoreaction [56].

#### NF-κB

Nuclear factor kappa B (NF-κB) is a transcription promoter and plays an important role in the PD-L2 regulation and the development of multiple types of HNSCC. Research showed that ribosomal receptor for activated C-kinase 1 (RACK1) mediates NF-κB to promote M2 macrophages in OSCC [57]. Moreover, NF-κB can mediate human papillomavirus (HPV)-induced HNSCC cancer progression [58]. In addition, activated NF-κB increased C-X-C motif chemokine ligand-1 (CXCL1) expression and secretion by cancer-associated fibroblasts (CAFs) to remodify TME in HNSCC [59]. Besides, HNSCC clinical samples and animal experiments showed that the PI3K/AKT axis can promote HNSCC survival via NF-κB [60]. NF-κB can bind to target gene sites through dimer formation to produce a marked effect [61]. Research has revealed that NF-κB transcription activates PD-L2 expression through a toll-like receptor (TRIF) and decreases TNF-α secretion in γδT cells [49].

#### GATA2

GATA2 is a hematopoietic transcription factor that contains zinc fingers (ZFs) that can bind to the PD-L2 gene. GATA2 can regulate normal hematopoiesis via stem cell maintenance and myeloid differentiation. GATA2 mutation not only contributes to hematological neoplasms but also drives tumour formation of HNSCC. GATA2 activates PD-L2 transcription through its transcription factor-binding site in the PD-L2 gene, thus inhibiting neoantigen-specific T-cell function in glioblastoma [62].

#### HOXC10

Homeobox C10 (HOXC10) is highly expressed in OSCC and induces the transformation from oral keratinocytes to OSCC [63]. In addition, HOXC10 contributes to invasion and metastasis by regulating Wnt signaling mediated EMT and confers to poor prognosis in HNSCC [63]. Recently, HOXC10 was identified to recognize the promoter region of the PD-L2 encoding gene and triggered PD-L2 expression in a transcription depending on the manner in the glioma [64].

#### ETV4

E26 transformation-specific variant transcription factor (ETV4) belongs to the ETS transcription factor family, which has been reported to play a role in promoting the proliferation, invasion, and drug resistance of various tumours [65]. ETV4 bound to the PD-L1–L2-SE region, which is dependent on the activation of the BRAF/TAK1/ERK axis through integrin αvβ3 stimulation, enhances the expression of PD-L2 to mediate immune escape in cancer cells [66].

#### OCT2

Octamer binding protein 2 (OCT2) regulates B-cell co-activation with OCA-B (also known as OBF-1 and BOB-1) if combined with DNA, and it can activate important factors such as STAT3, adenosine deaminase (ADA), and Myc [67]. A previous study has shown that OCT2 can increase PD-L2 localization in the cytomembrane of B cells when OCT2 is combined with the PD-L2 intro [68].

### Post-transcriptional regulation

Many non-coding RNAs, such as microRNAs (miRNAs) and long non-coding RNAs (lncRNAs), can activate as sponges for miRNAs. The abnormal expression patterns of non-coding RNAs correlate to tumorigenesis, tumour progression, and drug resistance in HNSCC. Hsa-mir-194-5p can suppress PD-L2 translation by combining with the 3’ untranslated region (3’UTR) of PD-L2 mRNA. Moreover, the lncRNA- PCED1B-AS1 promotes the PD-L2 translation by sponging and inhibiting hsa-mir-194-5p in hepatocellular carcinoma (HCC) [69]. PCED1B-AS1 can be transported by exosomes from HCC to immune cells to increase the occurrence of early apoptosis and decrease IL-2 secretion by T cells.

### Translation process

Variants of mRNA splicing give proteins different characteristics to promote the malignant phenotype of tumours, such as proliferation, migration, and invasion in HNSCC. There are several different splicing products of PD-L2 mRNA. The main type contains all six exons in its genome. Moreover, the other two types are different from the main type in terms of solubility. Soluble PD-L2 (sPD-L2) has a different location for diverse individuals and cell states that influence the immune response in epithelial ovarian cancer [70].

### Post-translational regulation

Epigenetic regulation can not only alter gene expression patterns but also participate in post-translational modification. Studies have confirmed that the PD-L2 protein undergoes glycosylation and ubiquitination modifications.

#### Glycosylation

Glycosylation plays an important role in the functional regulation of proteins. Most secreted proteins and membrane proteins undergo specific types of glycosylation modification. A specific N-linked glycosylation inhibitor- tunicamycin can result in a reduction in the molecular weight of PD-L2 by approximately 15 kDa in two colorectal cancer (CRC) cell lines [71]. Our group also found that PD-L2 was glycosylated by FUT8 in HNSCC, with N64, N157, N163, and N189 being identified as the four glycosylation sites of PD-L2. In addition, after glycosylation, PD-L2 combines with EGFR which provokes the EGFR/STAT3 signaling pathway and the subsequent transcription of FUT8 [23]. Intercepting FUT8 transcription by STAT3 inhibitor, Stattic, significantly deduce glycosylation PD-L2 protein level and thus decreases proliferation and resistance to cetuximab and EGFR inhibitors in HNSCC [23].

#### Ubiquitination

Ubiquitination modulates cell survival, differentiations and immune response by regulating protein stability and activity. We found that glycosylated PD-L2 affects its stability. Demonstrating that most protein stability in cells is mediated by the ubiquitin system, therefore, we investigated and verified that ubiquitination mediated PD-L2 degradation. The ubiquitination of PD-L2 can be recognized by endosomal sorting complexes required for transport (ESCRT) complexes, a vesicle transport complex necessary for the lysosomal degradation pathway, which can be degraded through lysosomal pathways. As a result, the glycosylated PD-L2 can inhibit the recognition of ESCRT, thereby inhibiting degradation and improving stability [23].

### Cell communication

#### LPS/CXCR3

Inhibiting C-X-C motif chemokine receptor 3 (CXCR3) through its inhibitor AMG487 can significantly inhibit the expression of PD-L2 under lipopolysaccharide (LPS) stimulation, which inhibits DC development. Subsequently, the expression of CD25, CD69, and IFN-γ production decreases, suggesting suppressed T-cell activation [72].

#### CCL2/CCR2

C-C motif chemokine ligand-2 (CCL2), through its receptor CCR2 (which is expressed on the surface of M2 TAMs), upregulates PD-L2 expression in M2 TAMs and reduces CD8 cytotoxic T lymphocyte (CTL)-mediated apoptosis of esophageal cancer cells (ESCCs), thereby causing immune escape in ESCC [^+^^73^].

#### IFN-α/ANO9

Anoctamin 9 (ANO9) belongs to the anoctamin family and is composed of transmembrane proteins that function as transporting chloride ions and crossing the membrane as a phospholipid scramblase. Interferon-alpha (IFN-α)- induced ANO9 promotes the expression of PD-L2 mRNA and PD-L2 membrane location, thereby mediating the cell cycle and increasing proliferation, migration, and invasion in GC [74].

#### FCP/TLR4

Fibrinogen cleavage products (FCPs) that induce allergic airway inflammation can stimulate TLR4 of mast cells to participate in PD-L2 DCs mediated T naive cell differentiation. The induction capability of PD-L2 DCs depends on the PD-L2 expression level in DCs [^+^^+^^75^].

### Others

#### GOLT1B

Golgi transport 1 B (GOLT1B) as a vesicle transporter that participates in protein trafficking in cytoplasm increases PD-L2 expression and facilitates PD-L2 membrane localization in colorectal cancer to promote apoptosis of T cells and decreases IFN-γ expression, which in turn promotes migration and invasion in CRC [30].

#### MMP9/13

Foreskin fibroblasts (FFs) can suppress the proliferation of CD3, CD4, and CD8 T cells via phytohemagglutinin (PHA)-stimulated T-cell apoptosis. Mechanistically, IL-1α, IFN-γ, transforming growth factor-beta (TGF-β), and TNF-α promote the expression of PD-L2 and combining capacity between PD-L2 and PD-1-Fc fusion protein in FFs. However, the PD-1-binding domain of PD-L2 can be cleaved by Matrix metalloproteinase 9/13 (MMP9/13) in FFs after exposure to γ radiation, thereby ameliorating T-cell inhibition [^+^^+^^+^^76^].

#### CAF

CAFs have shown the ability to interrupt the antigen presentation of CD8 + T cells, which may be mediated by some cytokines. For example, IFN-γ, produced by activated natural killer (NK) cells and T cells in TME, can directly induce upregulation of PD-L2 expression in HNSCC cells [77]. Also, TGF-β1, the expression of which is promoted by PD-L2 in CAFs, can inhibit the IFN-γ secretion of T cells. In contrast, CAFs can enhance IFN-γ secretion of T cells [78].

#### HPV and EBV

Viruses are known to drive HNSCC tumorigenesis, especially HPV [79] and Epstein–Barr virus (EBV). In HPV-positive (HPV) HNSCC, the HPV genome is integrated into the immune checkpoint gene PDCD1LG2, generating increased PD-L2 protein expression [^+^^80^]. Macrophages provoke the expression of PD-L2 in HPV HNSCC and lead to an immunosuppressive tumour environment. In addition, co-culturing of HPV HNSCC cells with fibroblasts can enhance PD-L2 expression in fibroblasts through Toll-like receptor 9 (TLR9), which can be blocked by the oligodeoxynucleotide TTAGGG in the absence of IFN-γ, TNF-α, and CD81 stimulation [^+^^81^, ^82^].

Nasopharyngeal carcinoma (NPC) is mainly caused by EBV infection, and clinical trials have shown that anti-PD-1 therapy can increase the one-year survival rate in NPC patients [83]. EBV-positive diffuse large B-cell lymphoma (DLBCL) cells have a high expression of PD-L2 and EBV miRNA. MiR-BHRF1-2-5p of EBV was able to bind with PD-L2 3’UTR to reduce PD-L2 expression, in order to drive B-cell differentiation [84].

## Future direction

**Fig. 3:**
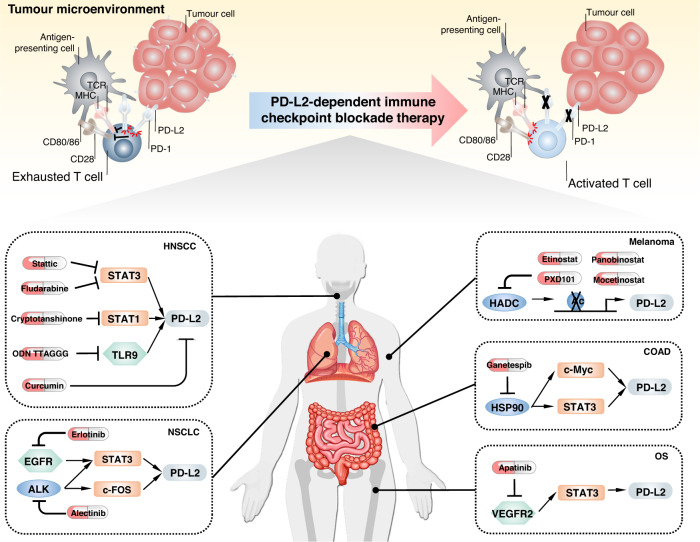
PD-L2-dependent immune checkpoint blockade therapy. PD-L2 is highly expressed in antigen-presenting cells and tumour cells, which binds PD-1 to inhibit CD80/86 to CD28 and MHC to TCR signal transduction. After PD-L2-dependent checkpoint blocked therapy, PD-L2 is exhausted, and CD80/86 and MHC activate T cells, thus playing a killing role in the tumour microenvironment (up section). Mechanism of small molecule inhibitors indirectly targeting PD-L2 in different solid tumours (down section).

PD-L2, an important PD-1 ligand, could be regulated in multiple ways and serve an irreplaceable role during the tumour immune escape process, eventually triggering tumorigenesis and tumour progression in various human cancers, including HNSCC. Thus, deeply studying of the PD-L2 regulatory mechanism is aimed to translate theoretical basic research into clinical application. Based on the recent finding in the mechanisms of the regulations of PD-L2 biological behavior, scientists have already designed several combination strategies targeting these regulations for cancer therapy and verified the theories in preclinical studies (Tables 2 and 3 and Fig. 3).

**Table 2:** PD-L2 blockade through indirect small molecule inhibitors or antibodies in tumours.

Regulation type	Compound	Target	PD-L2 expression	Cancer/cell type	Refs.
Epigenetic regulation	Panobinostat, Mocetinostat, Etinostat, PXD101	HDAC	Upregulation	Melanoma	[41]
	Ganetespib	HSP90	Downregulation	Colon adenocarcinoma	[43]
	Fludarabine	STAT1	Downregulation	Oral squamous cell carcinoma	[48]
	Cryptotanshinone	STAT3	Downregulation	Oral squamous cell carcinoma	[48]
	Stattic	STAT3	Downregulation	Head and neck squamous cell carcinoma	[21]
	Apatinib	VEGFR2	Downregulation	Osteosarcoma	[51]
	MDK-6314	STAT5	Downregulation	B cell	[53]
	Erlotinib	EGFR	Downregulation	Non-small cell lung cancer	[47]
	Alectinib	ALK	Downregulation	Non-small cell lung cancer	[47]
	Okadaic acid	OCT2	NA	B cell	[68]
	STAT5-IN-1	STAT5	Downregulation	Neutrophil	[54]
Extravesicular circulation	AMG487	CXCR3	Downregulation	Dendritic cell	[72]
	ODN TTAGGG	TLR9	Downregulation	Head and neck squamous cell carcinoma	[81]
	Curcumin	NA	Downregulation	Head and neck squamous cell carcinoma	[95]

*LBH589* panobinostat, *MGCD0103* mocetinostat, *HDAC* histone deacetylase, *OCT2* octamer binding protein 2, *HSP90* heat shock protein 90, *STAT1* signal transducer and activator of transcription 1, *STAT3* signal transducer and activator of transcription 3, *VEGFR2* vascular endothelial growth factor receptor-2, *STAT5* signal transducer and activator of transcription 5, *EGFR* epidermal growth factor receptor, *ALK* anaplastic lymphoma kinase, *CXCR3* C-X-C motif chemokine receptor 3, *TLR9* Toll-like receptor 9.

**Table 3:** Clinical value of PD-L2 attenuation in tumour cells and immune system.

Drugs	Target	Clinical trial/animal experiment	Cancer/cell type	Refs.
Panobinostat	HDAC	Animal experiment	Melanoma	[41]
Ganetespib	HSP90	Animal experiment	Colon adenocarcinoma	[43]
Stattic	STAT3	Animal experiment	Head and neck squamous cell carcinoma	[21]
Apatinib	VEGFR2	Animal experiment	Osteosarcoma	[51]
Diphtheria toxin	STAT3	Animal experiment	Mast cell, basophil	[75]
Curcumin	STAT3	Animal experiment	Head and neck squamous cell carcinoma	[95]

*HDAC* histone deacetylase, *HSP90* heat shock protein 90, *STAT3* signal transducer and activator of transcription 3, *VEGFR2* vascular endothelial growth factor receptor-2.

Some proposed combination therapies are being tested in clinical trials, including VEGFR2 inhibitor plus anti-PD-1 therapies (NCT03294694, NCT03147287, NCT03386929, and NCT03292250) [85–88], poly ADP-ribose polymerase (PARP) inhibitors plus PD-1/PD-L1 blockade therapies (NCT02657889, NCT03307785, and NCT03308942), and EGFR inhibitors plus anti-PD-1 therapies (NCT03082534, NCT02764593, and NCT02039674) [89, 90]. Researchers also tested other potential combinations; for instance, the curcumin plus anti-CTLA4 strategy has identified its safety and efficacy in mouse models, indicating that the successful combination strategy provides a scientific basis for further application in the clinical condition [91]. Until now, the theoretical research data were achieved under specific experimental in certain types of disease models, which may conflict with clinical treatment experience. For example, high EGFR-expressed triple-negative breast cancer (TNBC) mice can be benefited from immunotherapy. Therefore, identifying the right patient background is critical for testing in trials. Those results come from the specified type of disease model, so determining the benefit population background and predictive biomarkers are critical for successful clinical testing.

## Concluding remarks

Immunotherapy targeting PD-1 is a rapidly evolving field with the potential to benefit patients with HNSCC, as well as other advanced solid tumours. Nevertheless, due to the unfavorable clinical response, discoveries identifying novel predictive or therapeutic biomarkers are urgently encouraged. PD-L2, a PD-1 ligand, is generally expressed in multiple human cancers, including HNSCC, and serves as an independent predictor in anti-PD-1 immunotherapy. We propose that PD-L2 plays an important role in evading antitumor immunity as well as PD-L1, suggesting that PD-1/PD-L2 blockade must be considered for optimal immunotherapy in PD-L2-expressing cancers, such as HNSCC, RCC, and LUSC. However, the knowledge about the PD-L2 regulatory network is relatively ambiguous, and there is no clinical practice or trials about immunotherapy regimens against PD-L2 so far, both of which need in-depth research. This review provided a comprehensive study of the expression profile of PD-L2 and summarised mechanisms that regulate PD-L2 expression through multiple processes. Meanwhile, we emphasized the immune-dependent and independent functions of PD-L2 during anti-tumour immunotherapy.

Considering its predictive and therapeutic role, the clinical agents targeting PD-L2 may be beneficial for controlling tumour development. This strategy appears more achievable with the emergence of innovatively developed direct and/or nonspecific small molecule inhibitors of the PD-1/PD-L2 axis.

## References

[1] Brahmer JR, Tykodi SS, Chow LQ, Hwu WJ, Topalian SL, Hwu P (2012). Safety and activity of anti-PD-L1 antibody in patients with advanced cancer. N Engl J Med..

[2] Garon EB, Rizvi NA, Hui R, Leighl N, Balmanoukian AS, Eder JP (2015). Pembrolizumab for the treatment of non-small-cell lung cancer. N Engl J Med.

[3] Burtness B, Harrington KJ, Greil R, Soulieres D, Tahara M, de Castro G (2019). Pembrolizumab alone or with chemotherapy versus cetuximab with chemotherapy for recurrent or metastatic squamous cell carcinoma of the head and neck (KEYNOTE-048): a randomised, open-label, phase 3 study. Lancet..

[4] Ferris RL, Blumenschein G, Fayette J, Guigay J, Colevas AD, Licitra L (2016). Nivolumab for recurrent squamous-cell carcinoma of the head and neck. N Engl J Med..

[5] Jia L, Zhang Q, Zhang R (2018). PD-1/PD-L1 pathway blockade works as an effective and practical therapy for cancer immunotherapy. Cancer Biol Med..

[6] Lu J, T Li, Z Liao, H Yu, Y Zhao, H Wu, et al. The efficacies and biomarker investigations of antiprogrammed death-1 (anti-PD-1)-based therapies for metastatic bone and soft tissue sarcoma. Cancer Biol. Med. 2021;19:910. 10.20892/j.issn.2095-3941.2021.0270 PMC9257312 34817950

[7] Brahmer J, Reckamp KL, Baas P, Crino L, Eberhardt WE, Poddubskaya E (2015). Nivolumab versus docetaxel in advanced squamous-cell non-small-cell lung cancer. N Engl J Med..

[8] Mok TSK, Wu YL, Kudaba I, Kowalski DM, Cho BC, Turna HZ (2019). Pembrolizumab versus chemotherapy for previously untreated, PD-L1-expressing, locally advanced or metastatic non-small-cell lung cancer (KEYNOTE-042): a randomised, open-label, controlled, phase 3 trial. Lancet..

[9] Reck M, Rodriguez-Abreu D, Robinson AG, Hui R, Csoszi T, Fulop A (2016). Pembrolizumab versus chemotherapy for PD-L1-positive non-small-cell lung cancer. N Engl J Med..

[10] Motzer RJ, Robbins PB, Powles T, Albiges L, Haanen JB, Larkin J (2020). Avelumab plus axitinib versus sunitinib in advanced renal cell carcinoma: biomarker analysis of the phase 3 JAVELIN Renal 101 trial. Nat Med.

[11] Yearley JH, Gibson C, Yu N, Moon C, Murphy E, Juco J (2017). PD-L2 expression in human tumors: relevance to anti-PD-1 therapy in cancer. Clin Cancer Res.

[12] Matsubara T, Takada K, Azuma K, Takamori S, Toyokawa G, Haro A (2019). A clinicopathological and prognostic analysis of PD-L2 expression in surgically resected primary lung squamous cell carcinoma. Ann Surg Oncol.

[13] Shin SJ, Jeon YK, Kim PJ, Cho YM, Koh J, Chung DH (2016). Clinicopathologic analysis of PD-L1 and PD-L2 expression in renal cell carcinoma: association with oncogenic proteins status. Ann Surg Oncol.

[14] Zhang Y, Xu J, Hua J, Liu J, Liang C, Meng Q (2019). A PD-L2-based immune marker signature helps to predict survival in resected pancreatic ductal adenocarcinoma. J Immunother Cancer..

[15] Cancer Genome Atlas Research N, M Albert Einstein College of, S Analytical Biological, H Barretos Cancer, M Baylor College of, H Beckman Research Institute of City of, et al. Integrated genomic and molecular characterization of cervical cancer. Nature. 2017;543:378–84. 10.1038/nature21386 PMC5354998 28112728

[16] Mosconi C, de Arruda JAA, de Farias ACR, Oliveira GAQ, de Paula HM, Fonseca FP (2019). Immune microenvironment and evasion mechanisms in adenoid cystic carcinomas of salivary glands. Oral Oncol..

[17] Leng C, Li Y, Qin J, Ma J, Liu X, Cui Y (2016). Relationship between expression of PD-L1 and PD-L2 on esophageal squamous cell carcinoma and the antitumor effects of CD8(+) T cells. Oncol Rep..

[18] Qiao Y, Liu C, Zhang X, Zhou Q, Li Y, Xu Y (2021). PD-L2 based immune signature confers poor prognosis in HNSCC. Oncoimmunology..

[19] Huang KC, Chiang SF, Chen TW, Chen WT, Yang PC, Ke TW (2020). Prognostic relevance of programmed cell death 1 ligand 2 (PDCD1LG2/PD-L2) in patients with advanced stage colon carcinoma treated with chemotherapy. Sci Rep..

[20] Zhao SG, Lehrer J, Chang SL, Das R, Erho N, Liu Y (2019). The immune landscape of prostate cancer and nomination of PD-L2 as a potential therapeutic target. J Natl Cancer Inst.

[21] Sridharan V, Gjini E, Liao X, Chau NG, Haddad RI, Severgnini M (2016). Immune profiling of adenoid cystic carcinoma: PD-L2 expression and associations with tumor-infiltrating lymphocytes. Cancer Immunol Res.

[22] Umezu D, Okada N, Sakoda Y, Adachi K, Ojima T, Yamaue H (2019). Inhibitory functions of PD-L1 and PD-L2 in the regulation of anti-tumor immunity in murine tumor microenvironment. Cancer Immunol Immunother..

[23] Xu Y, Gao Z, Hu R, Wang Y, Wang Y, Su Z, et al. PD-L2 glycosylation promotes immune evasion and predicts anti-EGFR efficacy. J Immunother Cancer. 2021;9:e002699. 10.1136/jitc-2021-002699 PMC8547513 34697216

[24] Nakayama Y, Mimura K, Kua LF, Okayama H, Min AKT, Saito K (2020). Immune suppression caused by PD-L2 expression on tumor cells in gastric cancer. Gastric Cancer.

[25] Xu Y, Wu Y, Zhang S, Ma P, Jin X, Wang Z (2019). A tumor-specific super-enhancer drives immune evasion by guiding synchronous expression of PD-L1 and PD-L2. Cell Rep..

[26] Ran X, Xiao J, Zhang Y, Teng H, Cheng F, Chen H (2020). Low intratumor heterogeneity correlates with increased response to PD-1 blockade in renal cell carcinoma. Ther Adv Med Oncol.

[27] George S, Papanicolau-Sengos A, Lenzo FL, Conroy JM, Nesline M, Pabla S (2018). PD-L2 amplification and durable disease stabilization in patient with urothelial carcinoma receiving pembrolizumab. Oncoimmunology..

[28] Bakir B, Chiarella AM, PitarresiA JR, Rustgi K (2020). EMT, MET, plasticity, and tumor metastasis. Trends Cell Biol.

[29] Brabletz T, Kalluri R, NietoR MA, Weinberg A (2018). EMT in cancer. Nat Rev Cancer.

[30] Liu T, Liu B, Liu Y, Feng X, Jiang X, Long J (2021). Vesicle transporter GOLT1B mediates the cell membrane localization of DVL2 and PD-L2 and promotes colorectal cancer metastasis. Cancer Cell Int.

[31] Ren T, Zheng B, Huang Y, Wang S, Bao X, Liu K (2019). Osteosarcoma cell intrinsic PD-L2 signals promote invasion and metastasis via the RhoA-ROCK-LIMK2 and autophagy pathways. Cell Death Dis.

[32] Green MR, Monti S, Rodig SJ, Juszczynski P, Currie T, O’Donnell E (2010). Integrative analysis reveals selective 9p24.1 amplification, increased PD-1 ligand expression, and further induction via JAK2 in nodular sclerosing Hodgkin lymphoma and primary mediastinal large B-cell lymphoma. Blood..

[33] Ansell SM, Lesokhin AM, Borrello I, Halwani A, Scott EC, Gutierrez M (2015). PD-1 blockade with nivolumab in relapsed or refractory Hodgkin’s lymphoma. N Engl J Med..

[34] Yi M, Tan Y, Wang L, Cai J, Li X, Zeng Z (2020). TP63 links chromatin remodeling and enhancer reprogramming to epidermal differentiation and squamous cell carcinoma development. Cell Mol Life Sci.

[35] Pan Y, Song Y, Cheng L, Xu H, Liu J (2019). Analysis of methylation-driven genes for predicting the prognosis of patients with head and neck squamous cell carcinoma. J Cell Biochem.

[36] Vos L, Dietrich J, Strieth S, Bootz F, Dietrich D, Franzen A (2020). PD-1, CTLA4, PD-L1 and PD-L2 DNA methylation in papillary thyroid carcinoma. Immunotherapy..

[37] Masugi Y, Nishihara R, Hamada T, Song M, da Silva A, Kosumi K (2017). Tumor PDCD1LG2 (PD-L2) expression and the lymphocytic reaction to colorectal cancer. Cancer Immunol Res.

[38] Lingohr P, Dohmen J, Semaan A, Branchi V, Dietrich J, Bootz F (2019). Clinicopathological, immune and molecular correlates of PD-L2 methylation in gastric adenocarcinomas. Epigenomics..

[39] He L, Gao L, Shay C, Lang L, Lv F, Teng Y (2019). Histone deacetylase inhibitors suppress aggressiveness of head and neck squamous cell carcinoma via histone acetylation-independent blockade of the EGFR-Arf1 axis. J Exp Clin Cancer Res.

[40] Inaba K, Yashiro T, Hiroki I, Watanabe R, Kasakura K, Nishiyama C (2020). Dual roles of PU.1 in the expression of PD-L2: direct transactivation with IRF4 and indirect epigenetic regulation. J Immunol..

[41] Woods DM, Sodre AL, Villagra A, Sarnaik A, Sotomayor EM, Weber J (2015). HDAC inhibition upregulates PD-1 ligands in melanoma and augments immunotherapy with PD-1 blockade. Cancer Immunol Res..

[42] Tang YC, Hsiao JR, Jiang SS, Chang JY, Chu PY, Liu KJ (2021). c-MYC-directed NRF2 drives malignant progression of head and neck cancer via glucose-6-phosphate dehydrogenase and transketolase activation. Theranostics..

[43] Zavareh RB, Spangenberg SH, Woods A, Martinez-Pena F, Lairson LL (2021). HSP90 inhibition enhances cancer immunotherapy by modulating the surface expression of multiple immune checkpoint proteins. Cell Chem Biol.

[44] Cardenas MG, Oswald E, Yu W, Xue F, MacKerell AD, Melnick AM (2017). The expanding role of the BCL6 oncoprotein as a cancer therapeutic target. Clin Cancer Res.

[45] Worsham MJ, Lu M, Chen KM, Stephen JK, Havard S, Schweitzer VP (2012). Malignant and nonmalignant gene signatures in squamous head and neck cancer. J Oncol..

[46] Peng C, Hu Q, Yang F, Zhang H, Li F, Huang C (2019). BCL6-mediated silencing of PD-1 ligands in germinal center B cells maintains follicular T cell population. J Immunol..

[47] Shibahara D, Tanaka K, Iwama E, Kubo N, Ota K, Azuma K (2018). Intrinsic and extrinsic regulation of PD-L2 expression in oncogene-driven non-small cell lung cancer. J Thorac Oncol.

[48] Sudo S, Kajiya H, Okano S, Sasaki M, Katsumata Y, Ohno J (2020). Cisplatin-induced programmed cell death ligand-2 expression is associated with metastasis ability in oral squamous cell carcinoma. Cancer Sci.

[49] Xu P, Sun Z, Wang Y, Miao C (2015). Long-term use of indomethacin leads to poor prognoses through promoting the expression of PD-1 and PD-L2 via TRIF/NF-kappaB pathway and JAK/STAT3 pathway to inhibit TNF-alpha and IFN-gamma in hepatocellular carcinoma. Exp Cell Res..

[50] Horlad H, Ma C, Yano H, Pan C, Ohnishi K, Fujiwara Y (2016). An IL-27/Stat3 axis induces expression of programmed cell death 1 ligands (PD-L1/2) on infiltrating macrophages in lymphoma. Cancer Sci.

[51] Zheng B, Zhou C, Qu G, Ren C, Yan P, Guo W (2020). VEGFR2 promotes metastasis and PD-L2 expression of human osteosarcoma cells by activating the STAT3 and RhoA-ROCK-LIMK2 pathways. Front Oncol..

[52] Zerif E, FU Khan, AA Raki, V Lullier, D Gris, G Dupuis, et al. Elucidating the role of Ezh2 in tolerogenic function of NOD bone marrow-derived dendritic cells expressing constitutively active Stat5b. Int. J. Mol. Sci. 2020;21:6453. 10.3390/ijms21186453 PMC7554732 32899608

[53] McKay JT, Haro MA, Daly CA, Yammani RD, Pang B, Swords WE (2017). PD-L2 regulates B-1 cell antibody production against phosphorylcholine through an IL-5-dependent mechanism. J Immunol..

[54] Li C, Chen C, Kang X, Zhang X, Sun S, Guo F (2020). Decidua-derived granulocyte macrophage colony-stimulating factor induces polymorphonuclear myeloid-derived suppressor cells from circulating CD15+ neutrophils. Hum Reprod.

[55] Loke PJ, Allison P (2003). PD-L1 and PD-L2 are differentially regulated by Th1 and Th2 cells. Proc Natl Acad Sci USA..

[56] Huber S, Hoffmann R, Muskens F, Voehringer D (2010). Alternatively activated macrophages inhibit T-cell proliferation by Stat6-dependent expression of PD-L2. Blood..

[57] Dan H, Liu S, Liu J, Liu D, Yin F, Wei Z (2020). RACK1 promotes cancer progression by increasing the M2/M1 macrophage ratio via the NF-kappaB pathway in oral squamous cell carcinoma. Mol Oncol..

[58] Jin Y, Li Y, Wang X, Yang Y (2019). Secretory leukocyte protease inhibitor suppresses HPV E6-expressing HNSCC progression by mediating NF-kappaB and Akt pathways. Cancer Cell Int.

[59] Wei LY, Lee JJ, Yeh CY, Yang CJ, Kok SH, Ko JY (2019). Reciprocal activation of cancer-associated fibroblasts and oral squamous carcinoma cells through CXCL1. Oral Oncol..

[60] Lin J, Guan Z, Wang C, Feng L, Zheng Y, Caicedo E (2010). Inhibitor of differentiation 1 contributes to head and neck squamous cell carcinoma survival via the NF-kappaB/survivin and phosphoinositide 3-kinase/Akt signaling pathways. Clin Cancer Res.

[61] Morgan MJ, Liu ZG (2011). Crosstalk of reactive oxygen species and NF-kappaB signaling. Cell Res.

[62] Fu Y, Liu CJ, Kobayashi DK, Johanns TM, Bowman-Kirigin JA, Schaettler MO (2020). GATA2 regulates constitutive PD-L1 and PD-L2 expression in brain tumors. Sci Rep..

[63] Dai BW, Yang ZM, Deng P, Chen YR, He ZJ, Yang X (2019). HOXC10 promotes migration and invasion via the WNT-EMT signaling pathway in oral squamous cell carcinoma. J Cancer..

[64] Li S, Zhang W, Wu C, Gao H, Yu J, Wang X (2018). HOXC10 promotes proliferation and invasion and induces immunosuppressive gene expression in glioma. FEBS J..

[65] Keenan MM, Liu B, Tang X, Wu J, Cyr D, Stevens RD (2015). ACLY and ACC1 regulate hypoxia-induced apoptosis by modulating ETV4 via alpha-ketoglutarate. PLoS Genet.

[66] Ma P, Jin X, Fan Z, Wang Z, Yue S, Wu C, et al. Super-enhancer receives signals from the extracellular matrix to induce PD-L1-mediated immune evasion via integrin/BRAF/TAK1/ERK/ETV4 signaling. Cancer Biol. Med. 2022;19:669. 10.20892/j.issn.2095-3941.2021.0137 PMC9196059 34623791

[67] Hodson DJ, Shaffer AL, Xiao W, Wright GW, Schmitz R, Phelan JD (2016). Regulation of normal B-cell differentiation and malignant B-cell survival by OCT2. Proc Natl Acad Sci USA..

[68] Kaku HT, Rothstein L (2010). Octamer binding protein 2 (Oct2) regulates PD-L2 gene expression in B-1 cells through lineage-specific activity of a unique, intronic promoter. Genes Immun..

[69] Fan F, Chen K, Lu X, Li A, Liu C, Wu B (2021). Dual targeting of PD-L1 and PD-L2 by PCED1B-AS1 via sponging hsa-miR-194-5p induces immunosuppression in hepatocellular carcinoma. Hepatol Int..

[70] He XH, Liu Y, Xu LH, Zeng YY (2004). Cloning and identification of two novel splice variants of human PD-L2. Acta Biochim Biophys Sin..

[71] Wang H, Yao H, Li C, Liang L, Zhang Y, Shi H (2017). PD-L2 expression in colorectal cancer: independent prognostic effect and targetability by deglycosylation. Oncoimmunology..

[72] Qin C, Liu H, Tang B, Cao M, Yu Z, Liu B (2020). In vitro immunological effects of CXCR3 inhibitor AMG487 on dendritic cells. Arch Immunol Ther Exp..

[73] Yang H, Zhang Q, Xu M, Wang L, Chen X, Feng Y (2020). CCL2-CCR2 axis recruits tumor associated macrophages to induce immune evasion through PD-1 signaling in esophageal carcinogenesis. Mol Cancer..

[74] Katsurahara K, Shiozaki A, Kosuga T, Shimizu H, Kudou M, Arita T (2021). ANO9 regulates PD-L2 expression and binding ability to PD-1 in gastric cancer. Cancer Sci.

[75] Cho M, Lee JE, Lim H, Shin HW, Khalmuratova R, Choi G (2018). Fibrinogen cleavage products and Toll-like receptor 4 promote the generation of programmed cell death 1 ligand 2-positive dendritic cells in allergic asthma. J Allergy Clin Immunol.

[76] Dezutter-Dambuyant C, Durand I, Alberti L, Bendriss-Vermare N, Valladeau-Guilemond J, Duc A (2016). A novel regulation of PD-1 ligands on mesenchymal stromal cells through MMP-mediated proteolytic cleavage. Oncoimmunology..

[77] Chen SMY, Krinsky AL, Woolaver RA, Wang X, ChenJ Z, Wang H (2020). Tumor immune microenvironment in head and neck cancers. Mol Carcinog..

[78] Nazareth MR, Broderick L, Simpson-Abelson MR, Kelleher RJ, Yokota SJ, Bankert RB (2007). Characterization of human lung tumor-associated fibroblasts and their ability to modulate the activation of tumor-associated T cells. J Immunol..

[79] Viswanathan KP, Sadow M (2021). Somatostatin receptor 2 is highly sensitive and specific for Epstein-Barr virus-associated nasopharyngeal carcinoma. Hum Pathol..

[80] Succaria F, Kvistborg P, Stein JE, Engle EL, McMiller TL, Rooper LM (2021). Characterization of the tumor immune microenvironment in human papillomavirus-positive and -negative head and neck squamous cell carcinomas. Cancer Immunol Immunother..

[81] Baruah P, Bullenkamp J, Wilson POG, Lee M, KaskiI JC, Dumitriu E (2019). TLR9 mediated tumor-stroma interactions in human papilloma virus (HPV)-positive head and neck squamous cell carcinoma up-regulate PD-L1 and PD-L2. Front Immunol..

[82] Lechien JR, G Descamps, I Seminerio, S Furgiuele, D Dequanter, F Mouawad, et al. HPV involvement in the tumor microenvironment and immune treatment in head and neck squamous cell carcinomas. Cancers. 2020;12:1060. 10.3390/cancers12051060 PMC7281394 32344813

[83] Ma BBY, Lim WT, Goh BC, Hui EP, Lo KW, Pettinger A (2018). Antitumor activity of nivolumab in recurrent and metastatic nasopharyngeal carcinoma: an international, multicenter study of the Mayo Clinic Phase 2 Consortium (NCI-9742). J Clin Oncol..

[84] Cristino AS, Nourse J, West RA, Sabdia MB, Law SC, Gunawardana J (2019). EBV microRNA-BHRF1-2-5p targets the 3’UTR of immune checkpoint ligands PD-L1 and PD-L2. Blood..

[85] Lan C, Shen J, Wang Y, Li J, Liu Z, He M (2020). Camrelizumab plus apatinib in patients with advanced cervical cancer (CLAP): a multicenter, open-label, single-arm, phase II trial. J Clin Oncol.

[86] Fan Y, Zhao J, Wang Q, Huang D, Li X, Chen J (2021). Camrelizumab plus apatinib in extensive-stage SCLC (PASSION): a multicenter, two-stage, phase 2 trial. J Thorac Oncol.

[87] Cheng H, Zong L, Kong Y, Wang X, Gu Y, Cang W (2021). Camrelizumab plus apatinib in patients with high-risk chemorefractory or relapsed gestational trophoblastic neoplasia (CAP 01): a single-arm, open-label, phase 2 trial. Lancet Oncol.

[88] Xu J, Zhang Y, Jia R, Yue C, Chang L, Liu R (2019). Anti-PD-1 antibody SHR-1210 combined with apatinib for advanced hepatocellular carcinoma, gastric, or esophagogastric junction cancer: an open-label, dose escalation and expansion study. Clin Cancer Res..

[89] Gettinger S, Hellmann MD, Chow LQM, Borghaei H, Antonia S, Brahmer JR (2018). Nivolumab plus erlotinib in patients with EGFR-mutant advanced NSCLC. J Thorac Oncol.

[90] Yang JC, Gadgeel SM, Sequist LV, Wu CL, Papadimitrakopoulou VA, Su WC (2019). Pembrolizumab in combination with erlotinib or gefitinib as first-line therapy for advanced NSCLC with sensitizing EGFR mutation. J Thorac Oncol.

[91] Lim SO, Li CW, Xia W, Cha JH, Chan LC, Wu Y (2016). Deubiquitination and Stabilization of PD-L1 by CSN5. Cancer Cell.

[92] Shen T, Chen Z, Zhao ZJ, Wu J. Genetic defects of the IRF1-mediated major histocompatibility complex class I antigen presentation pathway occur prevalently in the JAK2 gene in non-small cell lung cancer. Oncotarget. 2017;8:60975–86. 10.18632/oncotarget.17689 PMC5617399 28977839

[93] Rover LK, Gevensleben H, Dietrich J, Bootz F, Landsberg J, Goltz D et al. PD-1 (PDCD1) promoter methylation is a prognostic factor in patients with diffuse lower-grade gliomas harboring isocitrate dehydrogenase (IDH) mutations. EBioMedicine. 2018;28:97–104. 10.1016/j.ebiom.2018.01.016 PMC5835568 29396294

[94] Hoffmann F, Zarbl R, Niebel D, Sirokay J, Fröhlich A, Posch C, et al. Prognostic and predictive value of PD-L2 DNA methylation and mRNA expression in melanoma. Clin. Epigenetics. 2020;12:94. 10.1186/s13148-020-00883-9 PMC7318478 32586358

[95] Liu L, Lim MA, Jung SN, Oh C, Won HR, Jin YL, et al. The effect of Curcumin on multi-level immune checkpoint blockade and T cell dysfunction in head and neck cancer. Phytomedicine. 2021;92:153758. 10.1016/j.phymed.2021.153758 34592487

